# Optical Attenuation Analysis of Biofilm Formation on Dental Prostheses Using Time-Domain Optical Coherence Tomography and Atomic Force Microscopy

**DOI:** 10.3390/dj14090563

**Published:** 2026-09-03

**Authors:** Maria Cecilia Galvez, Aaron Mharl Barlisan, Emmanuelle Hermosilla, Mark Nickole Tabafa, Ernest Macalalad, Jose Esmeria Jr., Edgar Vallar, Seiroh Okaneya, Jumar Cadondon, Tatsuo Shiina

**Affiliations:** 1 Environment and Remote Sensing Research (EARTH) Laboratory, Department of Physics, College of Science, De La Salle University, Manila 0922, Philippines; maria.cecilia.galvez@dlsu.edu.ph (M.C.G.); aaron_barlisan@dlsu.edu.ph (A.M.B.); emmanuelle_hermosilla@dlsu.edu.ph (E.H.); mark_nickole_tabafa@dlsu.edu.ph (M.N.T.); edgar.vallar@dlsu.edu.ph (E.V.); 2 Interdisciplinary Space Missions Development Division, Space Science Missions Bureau, Philippine Space Agency, Quezon City 1101, Philippines; ernest.macalalad@philsa.gov.ph; 3 Department of Physics, Mapua University, Manila 1002, Philippines; 4 Central Instrumentation Facility, Office of the Vice President for Research and Innovation, De La Salle University, Laguna Campus, Biñan City 4024, Philippines; jose.esmeria@dlsu.edu.ph; 5 Graduate School of Engineering, Chiba University, 1-33 Yayoicho, Chiba 263-8522, Japan; seiroh@phoenix-dent.co.jp (S.O.); shiina@faculty.chiba-u.jp (T.S.); 6 Phoenix-Dent Co., Ltd., 2649-3 Kameicho, Sano-shi 327-0024, Japan; 7 Division of Physical Sciences and Mathematics, College of Arts and Sciences, University of the Philippines Visayas, Miagao 5023, Philippines

**Keywords:** biofilm detection, optical attenuation, extinction coefficient, time-domain OCT, atomic force microscopy, dental prostheses

## Abstract

**Background/Objectives:** Dental biofilm formation on prosthetic materials contributes to oral diseases and material degradation. This pilot study evaluated the feasibility of combining time-domain optical coherence tomography (TD-OCT) and non-contact atomic force microscopy (NC-AFM) to characterize biofilm-associated optical and surface changes in dental prosthesis materials following antiseptic treatment. **Methods:** Acrylic resin, composite resin, and stainless-steel specimens were inoculated with *Streptococcus mutans* biofilms and exposed to water, Orahex, and Watsons mouthwash. TD-OCT was used to obtain extinction coefficient (EC) and optical thickness measurements, while NC-AFM was performed on Orahex-treated specimens to assess nanoscale surface morphology. EC distributions were analyzed descriptively from valid TD-OCT A-scans. **Results:** Within the examined specimens, EC distributions varied following biofilm formation and antiseptic treatment, with material-dependent responses. Composite resin exhibited the clearest reduction in EC following Watsons treatment, whereas acrylic resin and stainless-steel specimens showed more variable responses. Physical thickness remained relatively stable, while NC-AFM observations of the Orahex-treated specimens supported the TD-OCT findings by demonstrating corresponding surface morphological changes. **Conclusions:** This pilot study demonstrates the preliminary feasibility of integrating TD-OCT with NC-AFM for non-destructive evaluation of biofilm-associated optical and surface changes in dental prosthesis materials. The findings are descriptive and exploratory, highlighting the potential of TD-OCT for monitoring prosthetic materials while emphasizing the need for future studies incorporating independent biological replicates and complementary biological validation techniques.

## 1. Introduction

Dental health is a vital component of general well-being, encompassing physiological, aesthetic, and social aspects of human life. It directly affects mastication, speech, and appearance, influencing both nutrition and self-esteem [1]. Although dental diseases rarely pose immediate life-threatening risks, they can have significant social, psychological, and economic consequences. Maintaining oral health requires proper hygiene practices to prevent the accumulation of dental plaque and the progression of caries, both of which are closely associated with biofilm formation [2].

Oral biofilm plays a central role in the development of dental plaque and tooth decay. It is composed of diverse microbial communities attached to surfaces within the oral cavity, including teeth, gums, and prosthetic materials [3]. When the natural balance of this micro-ecosystem is disrupted—such as by the dominance of specific bacteria like *Streptococcus mutans*—the risk of dental caries increases [4]. If biofilms are not properly managed, they calcify into tartar or calculus within 24 h, becoming resistant to mechanical cleaning and contributing to periodontal diseases [5].

In cases of tooth loss or severe decay, dental prostheses—such as dentures, crowns, and bridges—restore oral function and aesthetics [6]. However, despite their inert composition, prosthetic surfaces are highly susceptible to biofilm colonization [7]. These biofilms not only compromise prosthesis longevity but also act as reservoirs for pathogenic microorganisms that can exacerbate oral and systemic diseases [8]. The surface properties of these materials, including their roughness, porosity, and surface charge, influence bacterial adhesion and retention [9,10]. Therefore, characterizing the biofilm–substrate interaction at multiple scales is essential for developing biofilm-resistant materials and effective disinfection strategies.

Imaging plays a pivotal role in dental science and biofilm research, allowing the visualization and quantification of microscopic structures that cannot be observed by the naked eye [11]. Optical Coherence Tomography (OCT) is a non-invasive imaging technique based on low-coherence interferometry that provides high-resolution, depth-resolved cross-sectional images of biological and synthetic samples [12]. Time-Domain OCT (TD-OCT), in particular, measures the interference between light backscattered from a sample and a reference mirror at varying optical path lengths [13,14]. The logarithmic decay of OCT signal intensity with depth can be modeled to derive the extinction coefficient (EC), which reflects the cumulative effect of scattering and absorption within the sample [15]. In biofilm-covered materials, variations in μ can therefore serve as an optical marker for bacterial accumulation, enabling quantitative detection without invasive sampling.

Complementary to OCT, Atomic Force Microscopy (AFM) provides nanoscale characterization of biofilm morphology and surface topography. Operating in non-contact mode, AFM captures 3D surface profiles without damaging the sample, offering insights into bacterial aggregation, surface roughness, and extracellular matrix distribution [16,17]. This combination of TD-OCT and AFM create a unique multimodal approach for correlating optical attenuation with nanoscale surface morphology—bridging optical physics with microbiological surface science.

Despite the established use of Spectral- and Swept-Source OCT systems in biomedical imaging, there is limited literature employing TD-OCT for quantitative biofilm analysis on dental prostheses. Existing studies predominantly focus on acrylic and composite resins [18], while stainless-steel prosthetic surfaces remain underexplored. Moreover, few investigations integrate OCT-based optical metrics with AFM validation to assess the effects of antiseptic treatments on biofilm-covered materials.

By combining optical modeling and nanoscale imaging, this research bridges the domains of applied optics and dental biomaterials, offering a novel, non-destructive framework for monitoring biofilm growth, antiseptic efficacy, and light–biofilm interactions. This interdisciplinary approach holds promise for advancing diagnostic imaging, material development, and clinical hygiene management in prosthodontics. Although OCT provides label-free, non-destructive assessment of biofilm-associated optical properties and AFM enables nanoscale characterization of surface morphology, neither technique directly measures bacterial viability or biofilm biomass [19]. Accordingly, the present study focuses on evaluating the capability of OCT-derived optical attenuation as an indicator of biofilm-associated structural changes rather than as a direct measurement of bacterial viability.

This study aims to address these gaps by utilizing a 1310 nm portable Time-Domain Optical Coherence Tomography system and Non-Contact Atomic Force Microscopy to investigate *Streptococcus mutans* biofilm formation and its reduction following antiseptic treatments on acrylic resin, composite resin, and stainless-steel dental substrates. The extinction coefficient derived from TD-OCT serves as a quantitative indicator of biofilm-induced optical attenuation, while AFM imaging validates surface morphology at the nanoscale.

## 2. Materials and Methods

### 2.1. Sample Preparation

**Figure 1:**
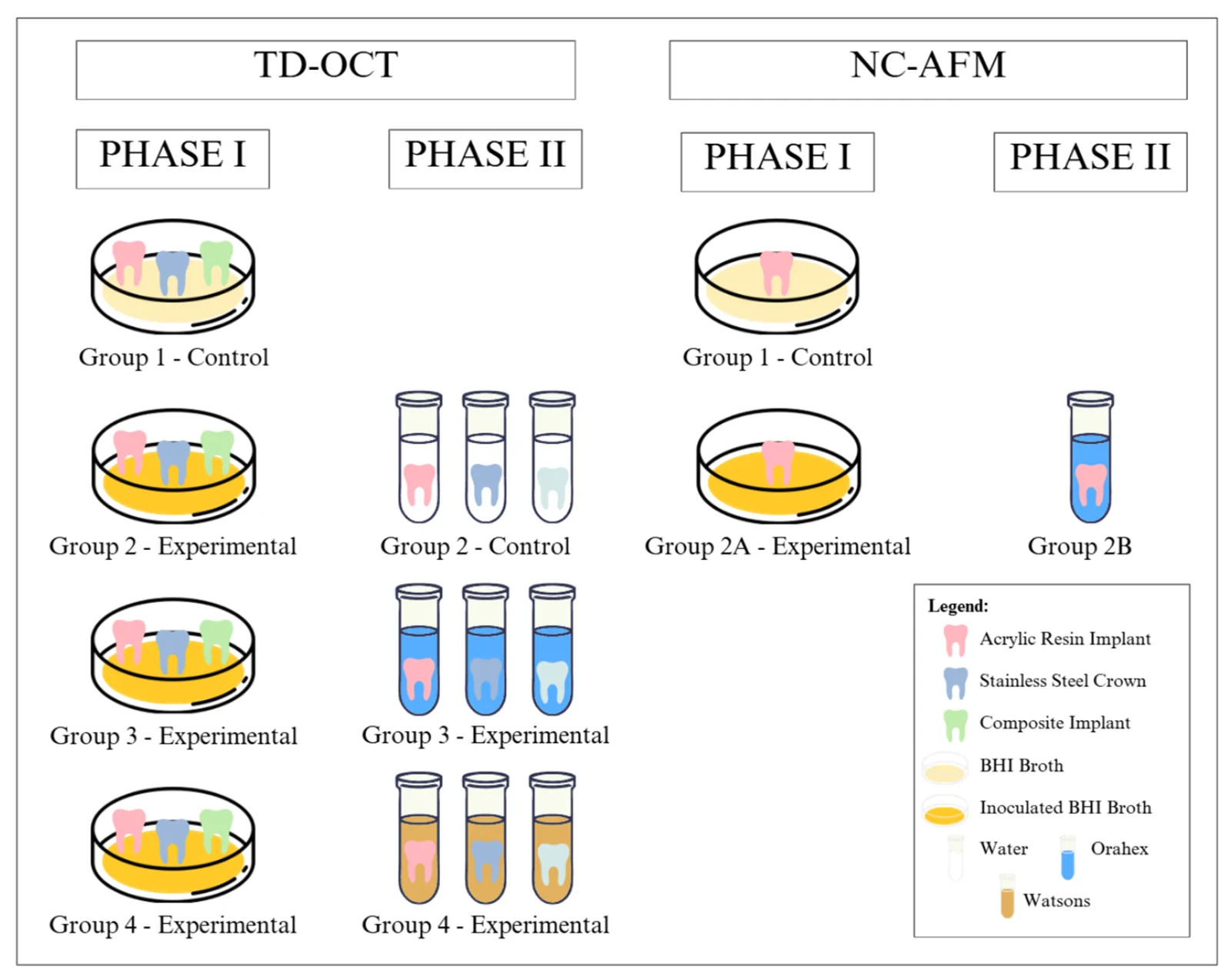
Representation of Control and Experimental groups used for TD-OCT and NC-AFM analyses.

A total of 21 anterior dental crowns were used as represented in Figure 1 and described in Table 1. All samples were sterilized in 70% ethanol before experiments. *S. mutans* (PNCM–BIOTECH) was cultured in Brain Heart Infusion (BHI) broth and incubated anaerobically at 37 °C using a candle-jar method for 48 h to promote biofilm formation.

Samples were divided into four TD-OCT groups and two NC-AFM groups. Control groups were immersed in sterile BHI broth, while experimental groups were inoculated with S. mutans suspension. Following biofilm growth (Phase 1), samples were treated with one of three antiseptic solutions (Phase 2): (a) distilled water, (b) 0.12% chlorhexidine gluconate (Orahex^®^) (Pharmahex, Inc., Pasig City, Philippines), or (c) Watsons^®^ Herbal Active Salt mouthwash (Watsons, Inc, Pasay City, Philippines). Each group contained three specimens in total: one acrylic resin specimen, one composite resin specimen, and one stainless-steel specimen. Thus, the physical specimen sample size was *n* = 1 for each material–condition combination. Multiple OCT A-scans were obtained from each specimen as technical and spatial measurements.

In the first imaging phase, the study utilized 4 sets of three anterior crowns, each set composed of different materials: acrylic resin, composite resin, and metal alloy substrates. Three of the first four groups are the experimental group, which underwent biofilm-promoting conditions, while the control group was submerged in uninoculated broth. The second phase is the antisepsis of the biofilm to measure the bacterial retention of each material. It utilized the experimental group of the first phase. The control group was exposed to water, while the experimental group was submerged in Orahex’s 0.12% Chlorhexidine gluconate oral solution and Watson’s Herbal Active Salt Mouthwash.

### 2.2. Monospecies Biofilm Synthesis

The bacterium *Streptococcus mutans* was selected as the test organism due to its dominance in the oral microbiome and its well-established role in dental caries formation. An agar slant culture of *S. mutans* was obtained from the Philippine National Collection of Microorganisms (PNCM–BIOTECH, University of the Philippines Los Baños).

Bacterial cultivation followed the protocol described by Cai et al. (2016), using Brain Heart Infusion (BHI) broth (HiMedia Laboratories, Mumbai, India) as the growth medium [20]. The medium was prepared by dissolving 18.5 g of BHI powder in 500 mL of distilled water in a sterile flask, covered with aluminum foil, and sterilized by autoclaving at 121 °C for 15 min. After cooling to ambient temperature, 250 mL of the sterilized broth was transferred aseptically to a secondary sterile flask for inoculation.

A single colony of *S. mutans* from the agar slant was transferred into the sterile BHI broth using an inoculating needle, and the culture was gently swirled to ensure uniform bacterial dispersion. Since *S. mutans* is a facultative anaerobe, incubation was performed in a low-oxygen environment generated using the candle-jar technique [21]. The inoculated flask and a lit candle were placed inside an airtight glass jar, which was sealed immediately; the candle’s extinguishing signified oxygen depletion and the establishment of a microaerophilic atmosphere. The culture was then maintained at room temperature for 48 h to allow bacterial proliferation and biofilm development.

Following incubation, approximately 10 mL of the bacterial suspension was dispensed into seven sterile Petri dishes, each containing three prosthetic samples representing the different test materials—acrylic resin, composite resin, and stainless steel. Control groups were prepared in parallel using the same protocol but with sterilized (uninoculated) BHI broth in place of the bacterial suspension.

### 2.3. TD-OCT Set-Up

#### Set-Up and Theoretical Perspective

**Figure 2:**
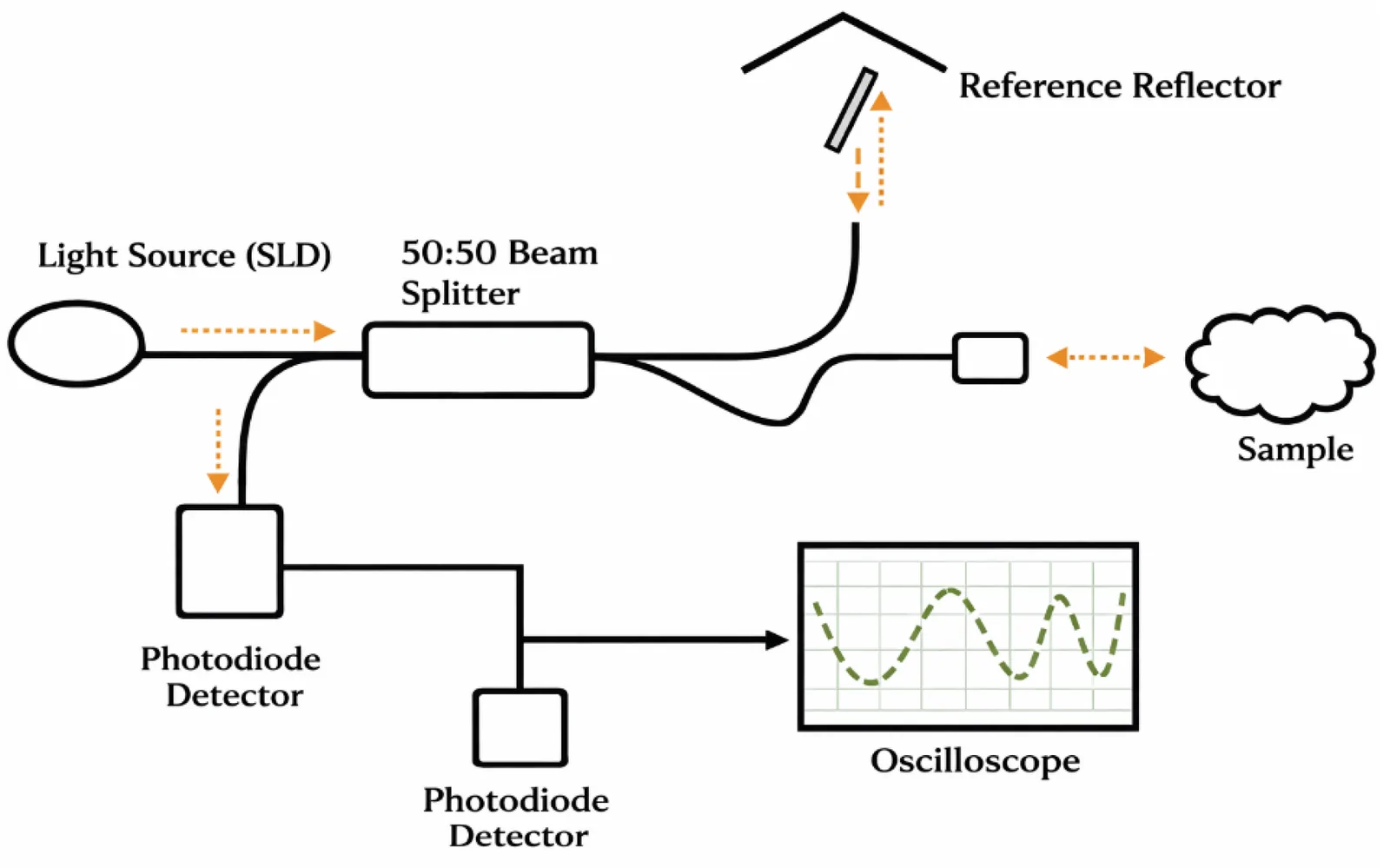
Schematic diagram of the portable 1310 nm TD-OCT system used in this study, reproduced from our previous methodological publication describing the same instrument configuration [24]. The earlier publication applied the system to plant leaf imaging, whereas the present study applies the identical TD-OCT platform to dental prosthesis characterization.

A custom-built portable TD-OCT system operating at a central wavelength of 1310 nm (axial resolution = 7 μm; lateral resolution = 3 μm) was used (Figure 2). The optical configuration and hardware are identical to those reported previously by our group [22,23,24], where the system was originally described and applied to plant leaf imaging. In the present study, the same TD-OCT platform was employed for the optical characterization of dental prosthesis materials. Signal averaging (128 waveforms) and background subtraction were implemented to improve signal-to-noise ratio. Samples were mounted on a 3D-printed holder to maintain alignment during scanning.

For each A-scan, OCT intensity $I\left(z\right)$ was modeled as

(1) $$I(z)=e^{-2\mu z},$$

where $I_{0}$ is the initial signal intensity and μ the extinction coefficient. Linearization via natural logarithm yields

(2) $$lnI(z)=lnI_{0}-2\mu z$$

allowing μ to be computed as $-\frac{1}{2}$× (slope) from the linear fit. A MATLAB R2026a routine was developed to apply a moving-mean filter, identify peak-valley regions in A-scans, perform log-linear fitting, and compute μ for each region. The detail of the developed algorithm is found in the Supplementary File S1. The mean extinction coefficient (EC) per sample was calculated as the average across all valid A-scans [25].

### 2.4. Non-Contact AFM Imaging

NC-AFM (Bruker) was used to verify surface morphology and quantify nanoscale roughness parameters: RMS roughness (Sq), mean roughness (Sa), and maximum height (Sz). Scans were performed on 25 × 25 μm and 15 × 15 μm areas of control, biofilm-contaminated, and antiseptic-treated acrylic resin samples. In this set-up, a flexible cantilever with a sharp probe mounted to a piezoelectric actuator raster-scans a surface to produce 3D topographic images at a subnanometer resolution. The tip interacts with the specimen surface through forces like van der Waals and electrostatic forces, causing the cantilever to deflect. This deflection is measured using a laser beam reflected onto a photodetector, creating a detailed surface map of the specimen [16,26].

### 2.5. Data Extraction, Preprocessing, and Analysis

For NC-AFM, three-dimensional topographic height profiles were analyzed to quantify surface morphology. Surface roughness parameters (Sa and Sq) and maximum surface height (Sz) were obtained from each scan area. The vertical distance between the highest peak and lowest valley within the scanned region (Sz) was used as an indicator of surface height variation, and the resulting values were averaged for each specimen.

For TD-OCT, thickness measurements were extracted from A-scan and B-scan datasets by identifying the optical path length difference between the biofilm–air interface and the biofilm–substrate interface. Optical thickness values were converted to physical thickness using the refractive index (*n* = 1.33) of the biofilm layer, as determined from literature-reported values for *S. mutans* biofilms [27]. Mean thickness per specimen was calculated by averaging measurements across all valid A-scans. For each material–condition combination, one physical specimen was analyzed. Multiple TD-OCT A-scans were acquired from different positions on each specimen. These A-scans represent repeated technical and spatial measurements within the same specimen and were not treated as independent biological or material replicates.

## 3. Results

### 3.1. TD-OCT Imaging

**Figure 3:**
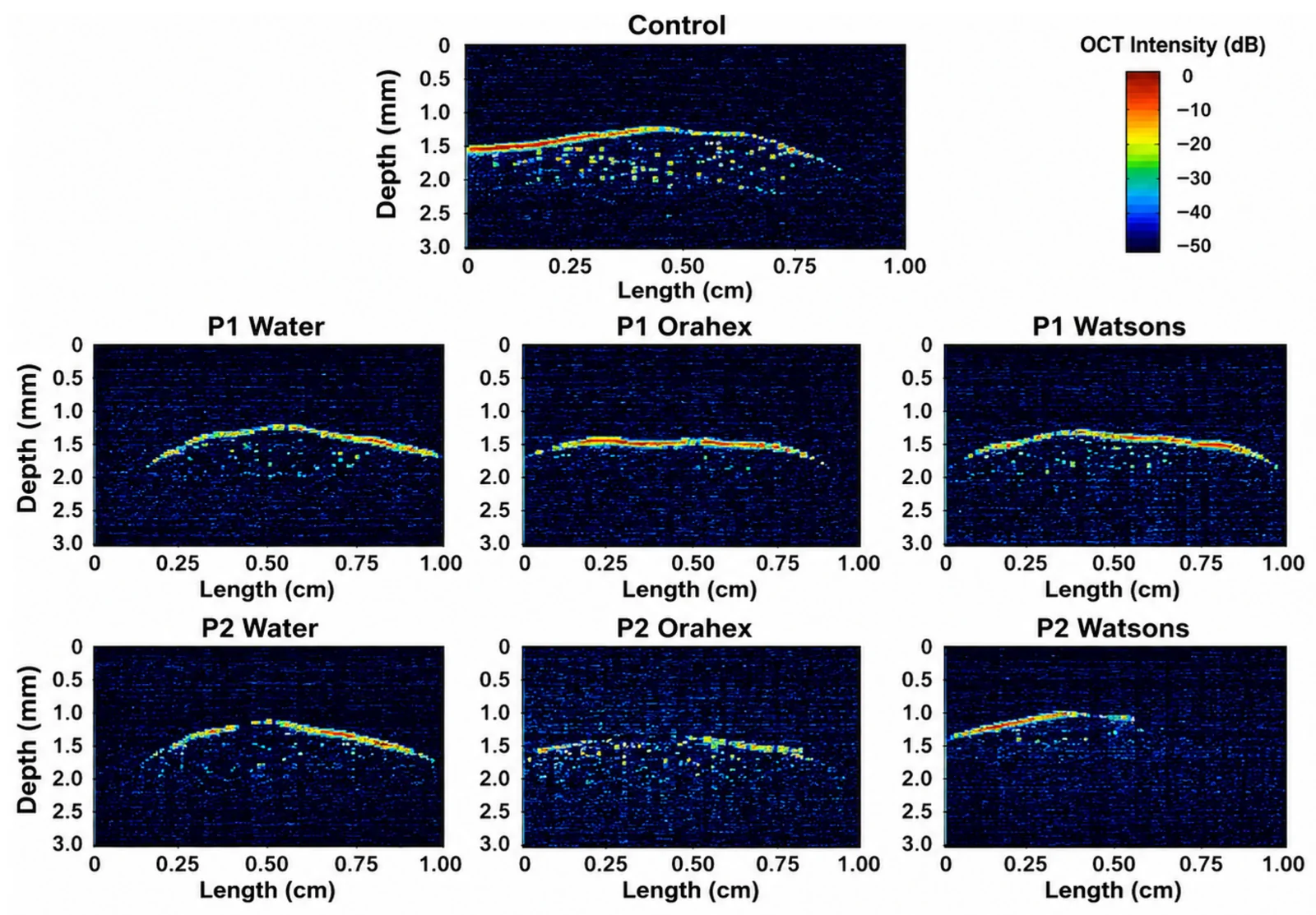
Representative TD-OCT B-scan images of acrylic resin specimens under the control, Phase 1 (P1: *S. mutans* biofilm formation), and Phase 2 (P2: post-antiseptic treatment) conditions. Antiseptic treatments consisted of distilled water, Orahex^®^ (0.12% chlorhexidine gluconate), and Watsons^®^ Herbal Active Salt mouthwash.

**Figure 4:**
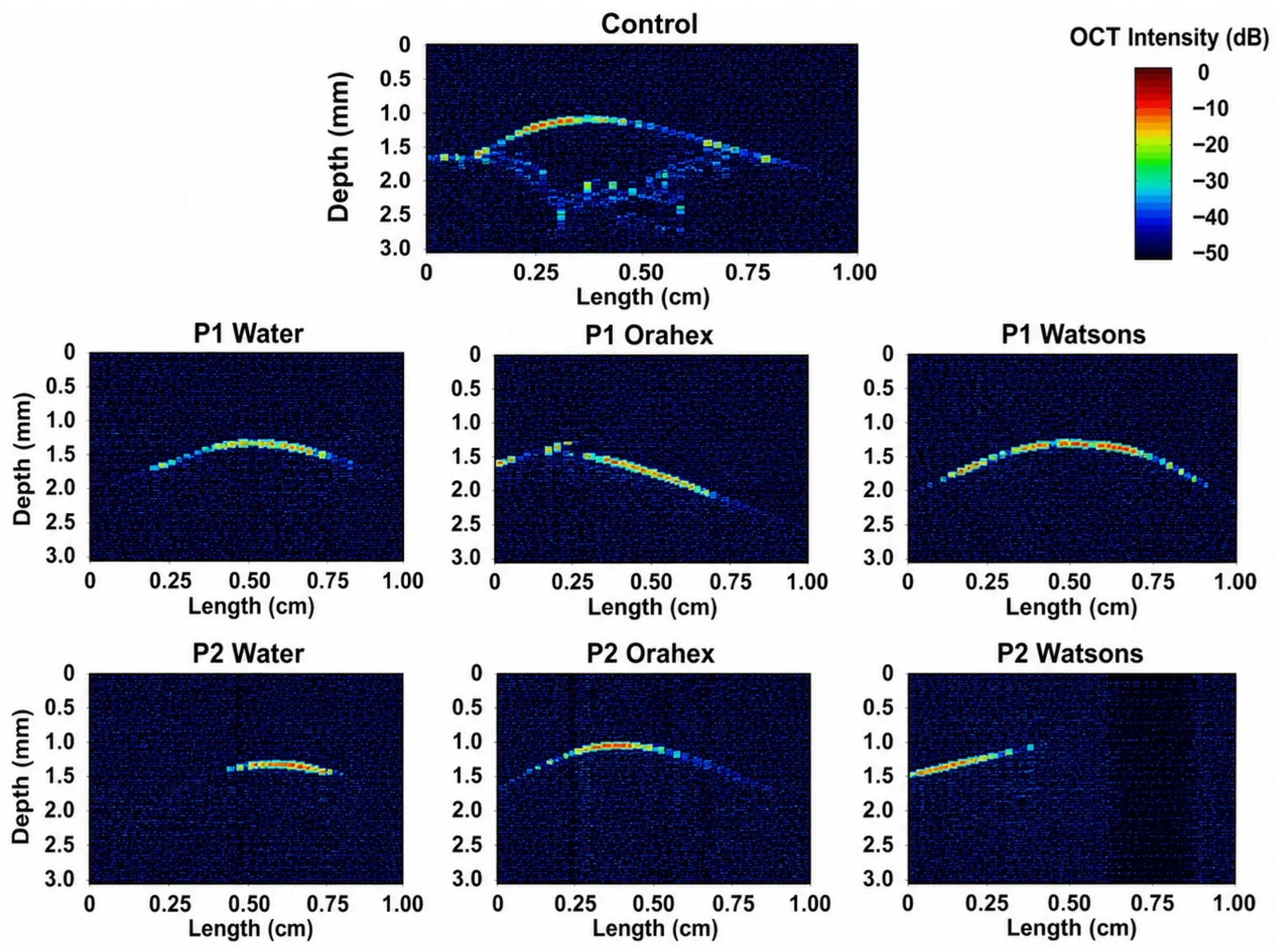
Representative TD-OCT B-scan images of composite resin specimens under the control, Phase 1 (P1: *S. mutans* biofilm formation), and Phase 2 (P2: post-antiseptic treatment) conditions. Antiseptic treatments consisted of distilled water, Orahex^®^ (0.12% chlorhexidine gluconate), and Watsons^®^ Herbal Active Salt mouthwash.

**Figure 5:**
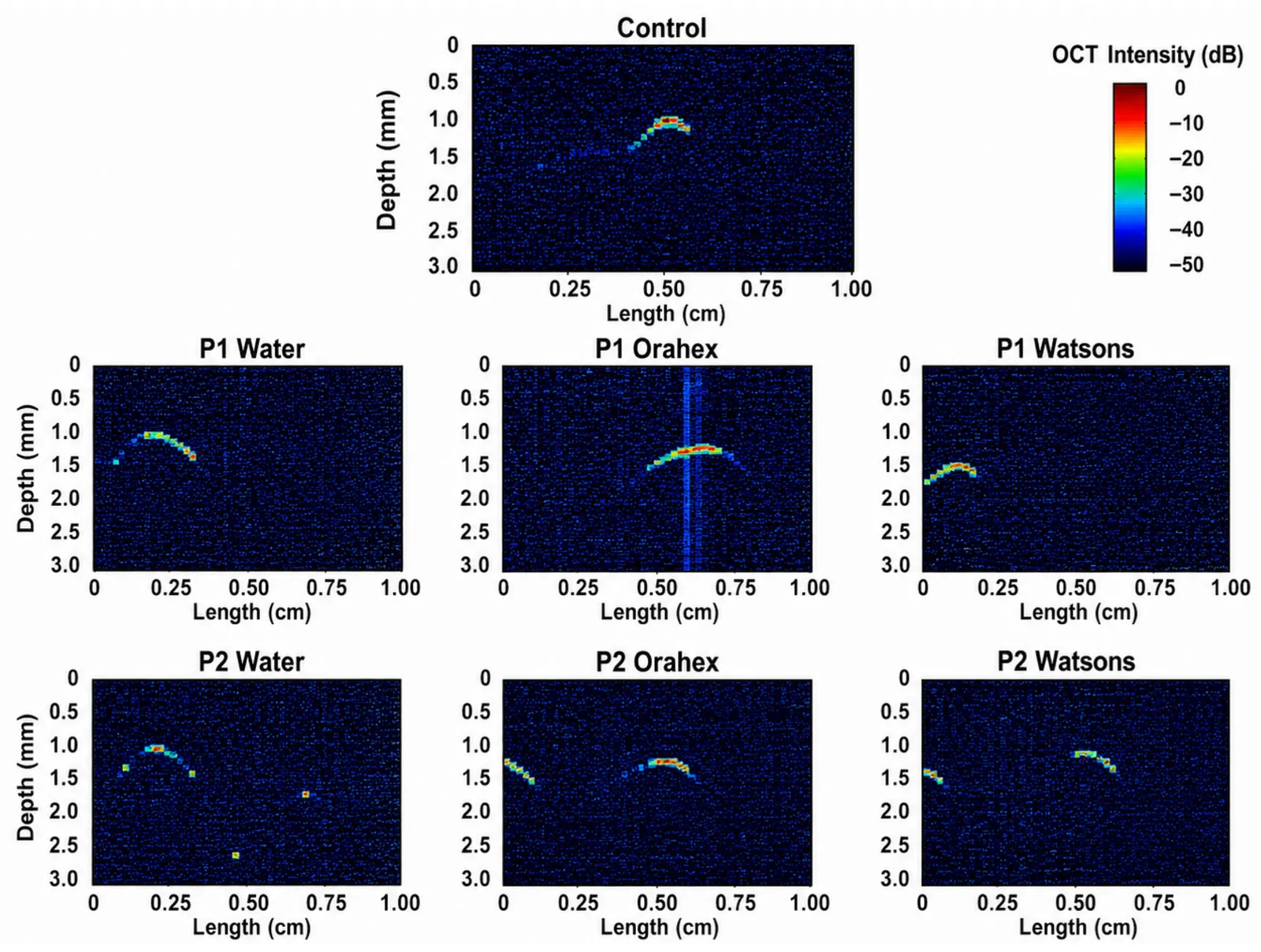
Representative TD-OCT B-scan images of stainless-steel specimens under the control, Phase 1 (P1: *S. mutans* biofilm formation), and Phase 2 (P2: post-antiseptic treatment) conditions. Antiseptic treatments consisted of distilled water, Orahex^®^ (0.12% chlorhexidine gluconate), and Watsons^®^ Herbal Active Salt mouthwash.

The TD-OCT B-scan images revealed distinct variations in optical response across the tested materials which include acrylic resin (Figure 3), composite resin (Figure 4), and stainless steel (Figure 5). For acrylic resin, the control samples exhibited a continuous, well-defined high-intensity arc, indicating a relatively smooth and homogeneous surface interface. Subsurface regions showed moderate scattering, suggesting limited internal heterogeneity. Under P1 and P2 treatments, slight disruptions in the continuity of the reflective band were observed, with P2 showing more pronounced irregularities. These changes imply increasing surface roughness or microstructural alteration induced by treatment, particularly when exposed to Orahex and Watsons solutions.

In contrast, composite resin demonstrated a less uniform and more diffuse reflective profile, even in control conditions. The B-scans showed increased speckle noise and scattering, consistent with its heterogeneous composition due to filler particles [28]. With P1 and P2 treatments, the reflective arcs became increasingly fragmented and attenuated, indicating enhanced internal scattering and possible degradation of the resin matrix–filler interface. Among the exposure media, Watsons and Orahex produced more noticeable disruptions compared to water, suggesting stronger interactions with the composite structure.

For stainless steel, the B-scans were characterized by high-intensity, localized reflections with minimal depth penetration, attributable to its reflectivity and opacity. Unlike the resin-based materials, subsurface features were largely absent. However, variations in the shape and continuity of the reflected signal were still evident across treatments. P1 and P2 conditions showed slight distortions and discontinuities, which may be associated with surface modifications such as roughening or residue deposition. The effect of exposure media was less pronounced compared to the resins but still detectable in the form of signal irregularities [29].

Water exposure maintained relatively clearer and more stable interface signals, whereas Orahex and Watsons solutions contributed to increased signal variability and scattering, particularly under P2 conditions. This trend suggests that chemical exposure plays a role in modifying surface morphology and optical properties.

#### 3.1.1. EC Variations

**Figure 6:**
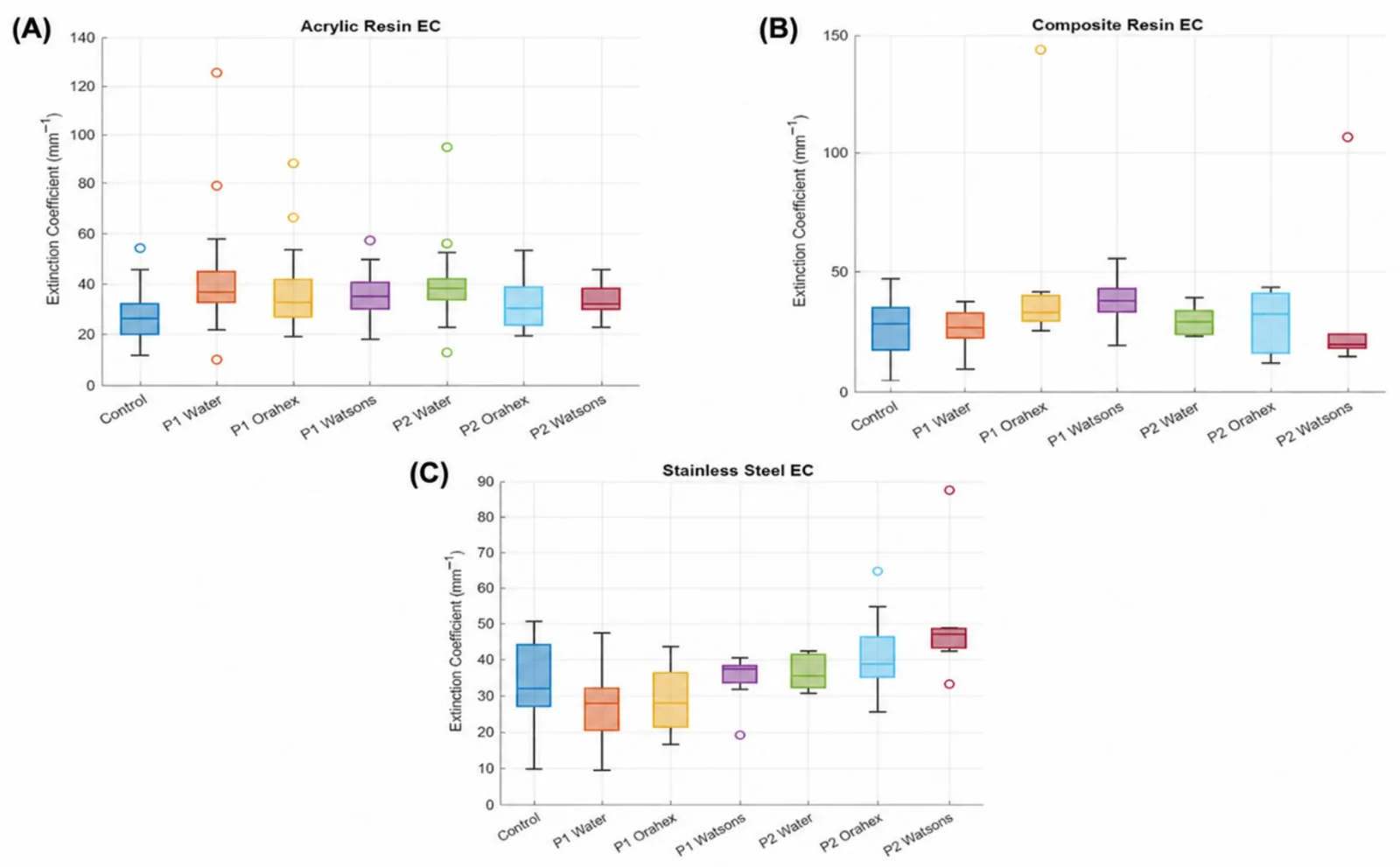
Distribution of extinction coefficient measurements obtained from multiple valid TD-OCT A-scans acquired from one specimen for (**A**) Acrylic resin, (**B**) Composite resin, and (**C**) Stainless Steel. The boxplots represent within-specimen spatial and technical variability and do not represent variability among independent specimens. Boxes indicate the interquartile range, center lines indicate the median, whiskers represent values within 1.5 times the interquartile range, and circle denote values outside this range. Accordingly, these boxplots are presented for descriptive purposes only and should not be interpreted as inferential statistical comparisons among the experimental groups or treatment conditions.

The TD-OCT-derived EC values revealed differences in optical attenuation among acrylic resin, composite resin, and stainless steel after exposure to the test solutions (Figure 6). Higher EC values indicate greater light scattering and attenuation, which are often associated with increased structural heterogeneity, surface changes, or material degradation [30,31].

The mean EC for each specimen was calculated from all valid A-scans that satisfied the preprocessing and logarithmic curve-fitting criteria. The number of valid A-scans varied among specimens depending on signal quality and successful peak–valley identification, ranging from 6 to 34 valid A-scans per specimen. Within the specimens examined, the acrylic resin measurements showed the widest distribution of EC values across sampled A-scan locations. Acrylic resin has median EC values generally ranging from 25–40 mm^−1^. The control group showed a median EC of approximately 26 mm^−1^, while the treated groups increased to approximately 35–39 mm^−1^. Several high outliers were observed, reaching 125 mm^−1^ in P1 Water and 95 mm^−1^ in P2 Water, suggesting localized regions of increased scattering and non-uniform structural changes. Composite resin showed moderate EC values, with medians ranging from approximately 22–38 mm^−1^. The highest median was observed in P1 Watsons (~38 mm^−1^), whereas P2 Watsons (~21 mm^−1^) exhibited the lowest value. Although most groups remained relatively stable, extreme outliers of approximately 145 mm^−1^ (P1 Orahex) and 107 mm^−1^ (P2 Watsons) indicate localized alterations within the material. The composite resin specimen exhibited a narrower EC distribution than the acrylic resin specimen under several examined conditions. This observation is specimen-specific and requires confirmation using independently replicated samples.

For stainless steel, the EC values were generally lower and more consistent, with median values ranging from approximately 28–46 mm^−1^. The highest attenuation was observed in P2 Watsons (~46 mm^−1^), compared with the control (~32 mm^−1^). Outliers reaching 65 mm^−1^ (P2 Orahex) and 88 mm^−1^ (P2 Watsons) suggest localized surface modifications, likely related to changes in surface roughness or oxide layer formation rather than bulk structural changes. The observed differences in extinction coefficient among A-scan locations suggest spatial variation in optical attenuation within the examined specimens. Because the extinction coefficient represents the cumulative effects of optical scattering and absorption, the measured variations may arise from multiple contributing factors, including biofilm formation, residual biofilm following antiseptic treatment, changes in surface roughness, water absorption, and treatment-induced microstructural modifications of the substrate [32,33]. Therefore, the EC should be interpreted as an integrated optical parameter rather than as an exclusive indicator of biofilm presence.

#### 3.1.2. Optical Thickness

Based on the TD-OCT B-scan images, the optical thickness was measured based on the refractive index and the two consecutive A-scan measurements [22,25] as provided in Table 2. The physical thicknesses of all specimens ranged from approximately 0.86 to 1.24 μm. The acrylic resin specimen exhibited relatively uniform thicknesses (1.08 ± 0.08 μm), while composite resin showed the largest variation (1.04 ± 0.11 μm), mainly due to the lower thickness observed in the P2 Orahex group. Stainless steel displayed the greatest thickness under P1 Watsons (1.24 μm) and an overall mean thickness of 1.09 ± 0.11 μm.

The relatively small variations in physical thickness compared with the larger variations observed in the extinction coefficient indicate that the treatment solutions had a stronger influence on the optical attenuation properties than on specimen thickness. This finding further supports the sensitivity of TD-OCT-derived extinction coefficient measurements for detecting treatment-induced modifications in dental materials.

### 3.2. AFM Morphology and Roughness

**Figure 7:**
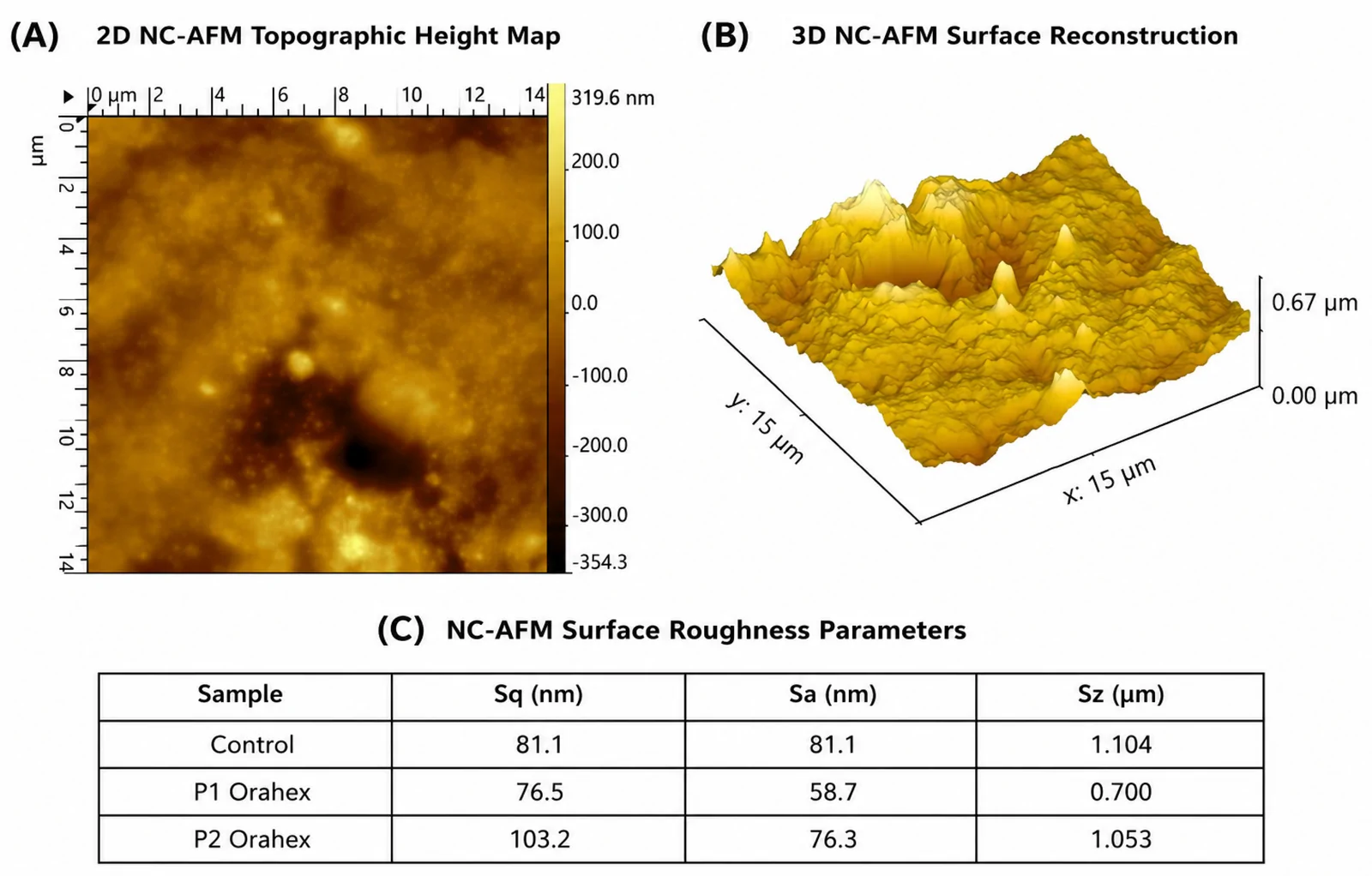
Non-contact atomic force microscopy (NC-AFM) characterization of Orahex-treated dental material surfaces. (**A**) Two-dimensional topographic height map showing nanoscale surface features across a 15 × 15 μm scan area. (**B**) Three-dimensional surface reconstruction demonstrating the spatial distribution of peaks and valleys with a maximum height variation of approximately 0.67 μm. (**C**) Quantitative surface roughness parameters including root mean square roughness (Sq), arithmetic mean roughness (Sa), and maximum surface height (Sz) for the Control, P1 Orahex, and P2 Orahex groups.

Surface characterization using NC-AFM was performed only on the Orahex-treated specimens (Figure 7) because Orahex produced notable changes in the TD-OCT-derived extinction coefficient distributions. Consequently, the AFM observations presented in this study are limited to the Orahex treatment and should not be interpreted as direct validation of the water- or Watsons-treated specimens.

The NC-AFM analysis revealed that the control sample exhibited the largest surface height variation (Sz = 1.104 µm), indicating the presence of pronounced surface peaks and valleys. Following P1 Orahex treatment, the maximum height decreased substantially to 0.700 µm, suggesting a smoother and more uniform surface. In contrast, P2 Orahex exhibited the highest RMS roughness (Sq = 103.2 nm) and a maximum height of 1.053 µm, indicating the reappearance of larger topographical features and increased surface heterogeneity. The reduction in surface height variation observed in P1 Orahex corresponds well with the lower extinction coefficient variability observed from TD-OCT measurements, suggesting a decrease in optical scattering due to surface smoothing. Conversely, the increased roughness and height variation in P2 Orahex may contribute to enhanced light scattering and attenuation, which is reflected by the higher extinction coefficient values measured using TD-OCT.

## 4. Discussion

### 4.1. TD-OCT Images and Its Optical Responses to Dental Prostheses

The observed EC responses differed among the examined specimens and treatment conditions. Composite resin showed the clearest reduction in EC following Watsons treatment (Figure 4), whereas acrylic resin exhibited broader EC distributions across treatment groups (Figure 3) and the stainless-steel specimen showed less consistent responses (Figure 5), likely reflecting the combined influence of high surface reflectivity, multiple scattering, and material-specific optical properties [34]. The extinction coefficient analysis further supported these observations. Acrylic resin exhibited the greatest variability in EC values, indicating higher susceptibility to treatment-induced changes. Composite resin showed intermediate behavior, while stainless steel exhibited the most consistent EC distributions. Increased EC values correspond to greater optical attenuation and scattering, which are commonly associated with increased surface roughness, water absorption, microstructural heterogeneity, or degradation of the material matrix. These observations suggest that Orahex and Watsons solutions had a stronger influence on the optical properties of the materials than water exposure alone.

In contrast, optical thickness measurements showed relatively small variations among treatment groups (Figure 6). The thickness values remained within a narrow range for all materials despite noticeable changes in extinction coefficient. This indicates that the mouthwash solutions primarily affected optical attenuation properties rather than causing substantial dimensional changes. Therefore, EC appears to be a more sensitive parameter for detecting treatment-induced modifications than thickness measurements alone.

The NC-AFM results provided additional evidence of surface modifications following Orahex exposure (Figure 7A,B). The reduction in roughness and surface height variation observed in the P1 Orahex group suggests a smoother and more homogeneous surface, whereas the increased roughness observed in the P2 Orahex group indicates the development of larger surface features and greater heterogeneity (Figure 7C). These nanoscales changes correspond well with the variations observed in extinction coefficient measurements, supporting the relationship between surface morphology and optical scattering behavior. The interpretation of the extinction coefficient requires consideration of multiple contributing mechanisms. During the initial biofilm formation stage (P1), increases in EC are primarily attributed to the additional optical scattering introduced by the developing *S. mutans* biofilm. Following antiseptic treatment (P2), however, the measured EC likely reflects the combined effects of residual biofilm, changes in biofilm architecture, surface roughness, water absorption, and possible chemical or microstructural modifications of the underlying dental material [35,36]. Consequently, the observed EC variations cannot be attributed exclusively to either biofilm removal or substrate modification but instead represent the cumulative optical response of the biofilm–material interface [37].

### 4.2. Clinical Implications and Limitations of TD-OCT Attenuation Analysis

The TD-OCT analysis demonstrated its effectiveness as a non-destructive technique for evaluating changes in the optical and structural properties of dental materials following exposure to different mouthwash solutions. By measuring the EC and optical thickness, TD-OCT provided quantitative information regarding light attenuation, scattering behavior, and subsurface structural integrity. Variations in EC among the acrylic resin, composite resin, and stainless-steel samples indicate that the tested solutions influenced the materials differently, reflecting changes in their optical homogeneity and surface condition.

In dental applications, the extinction coefficient is particularly important because it is sensitive to alterations in surface roughness, porosity, microstructural heterogeneity, and water absorption [38,39]. The higher EC values observed in some treated groups suggest increased optical scattering, which may be associated with material degradation or surface modifications induced by prolonged exposure to oral care products. For the Orahex-treated specimens, the agreement between TD-OCT attenuation measurements and NC-AFM surface morphology supports the capability of TD-OCT to detect subtle treatment-associated surface changes. Because AFM characterization was not performed for the water- or Watsons-treated groups, this multimodal correspondence should not be generalized beyond the Orahex condition. The agreement observed between TD-OCT and AFM for the Orahex-treated specimens highlights the capability of TD-OCT to detect subtle material changes without physically altering the specimen. Although TD-OCT is highly sensitive to changes in optical attenuation, it cannot independently distinguish whether the observed scattering originates from biofilm thickness, residual biomass, changes in surface roughness, water uptake, or chemical alterations of the substrate [40]. Therefore, the extinction coefficient should be interpreted as an integrated optical parameter reflecting the combined response of the biofilm and the underlying material. Additional biological and physicochemical characterization techniques would further improve discrimination between these individual mechanisms.

TD-OCT offers significant advantages for dental biomaterials research because it enables rapid, real-time assessment of restorative materials, implants, and prosthetic components [41]. The technique can identify early structural changes before visible deterioration occurs, making it valuable for predicting the long-term performance and durability of dental restorations. Therefore, the observed changes in extinction coefficient and optical thickness demonstrate that TD-OCT is a sensitive and reliable tool for monitoring the effects of chemical exposure on dental materials and for supporting the development of more durable restorative systems.

While the OCT attenuation measurements and AFM surface characterization consistently demonstrated changes following *S. mutans* biofilm formation and antiseptic treatment, the present study did not include direct microbiological quantification of bacterial viability or biofilm biomass. AFM primarily provides nanoscale topographical information and therefore cannot independently distinguish viable from non-viable bacterial cells or quantify total biomass [42]. Likewise, OCT attenuation reflects changes in optical scattering resulting from alterations in the biofilm structure and material interface rather than providing direct biological measurements.

A major limitation of this study is the absence of independent specimen replication within each material–condition combination. Although multiple A-scans were acquired from each specimen, these measurements are nested within the same physical sample and therefore represent technical and spatial variability rather than independent experimental replicates. Consequently, the EC distributions cannot be used to estimate between-specimen variability or to establish statistically generalizable treatment effects. The comparisons among materials and treatment conditions should therefore be interpreted as exploratory, specimen-level observations [43]. Future studies should include multiple independently prepared specimens for every material and treatment condition and apply statistical models that account for the hierarchical structure of A-scans nested within specimens.

To minimize potential confounding factors, all experimental groups were prepared using identical substrate materials, standardized biofilm culture conditions, identical OCT acquisition parameters, and the same image-processing procedure. Consequently, the principal experimental variable was the presence or reduction of the *S. mutans* biofilm following antiseptic treatment. Nevertheless, additional biological validation using complementary techniques such as colony-forming unit (CFU) counting, crystal violet biomass assays, live/dead fluorescence staining, scanning electron microscopy (SEM), or confocal laser scanning microscopy would further strengthen the biological interpretation of the OCT attenuation measurements and help distinguish biofilm-specific optical changes from any residual influence of the substrate material or antiseptic solution [44]. These multimodal validation approaches will be incorporated in future investigations.

An important limitation of the present pilot study is that each material–condition combination was represented by a single physical specimen. Although multiple valid TD-OCT A-scans were acquired from each specimen to characterize within-specimen optical variability, these measurements do not constitute independent biological or material replicates. Consequently, the observed extinction coefficient distributions should be interpreted as descriptive observations rather than statistically generalizable treatment effects. Future studies should include multiple independently prepared specimens for each material and treatment condition to enable rigorous inferential statistical analyses, account for specimen-to-specimen variability, and establish the reproducibility and generalizability of the present findings. Expanding the experimental design in this manner will also facilitate more comprehensive multimodal validation of TD-OCT measurements through complementary biological assays and NC-AFM characterization across all treatment groups.

## 5. Conclusions

This pilot study demonstrates the preliminary feasibility of TD-OCT and NC-AFM for evaluating the effects of commercially available mouthwash solutions on acrylic resin, composite resin, and stainless-steel dental materials. TD-OCT provided non-destructive assessment of the optical properties through EC and optical thickness measurements, while NC-AFM enabled nanoscale characterization of surface roughness and topographical changes. The TD-OCT results revealed that exposure to the tested mouthwash solutions influenced the optical attenuation characteristics of all materials, with acrylic resin exhibiting the greatest variability in extinction coefficient values, followed by composite resin, while stainless steel showed the most stable response. Despite these variations in optical attenuation, the measured optical thicknesses remained relatively consistent, ranging from approximately 0.86 to 1.24 μm. This indicates that the observed changes were primarily associated with modifications in light scattering and material heterogeneity rather than significant dimensional alterations.

NC-AFM analysis of the Orahex-treated specimens further confirmed the presence of surface modifications at the nanometer scale. P1 Orahex treatment resulted in reduced surface roughness and maximum surface height variation compared with the control, suggesting a smoother and more uniform surface. P2 Orahex exhibited increased RMS roughness and surface height variation, indicating the development of greater surface heterogeneity. These findings support the TD-OCT observations, where changes in optical attenuation were linked to alterations in surface morphology and scattering behavior. The combined results suggest that mouthwash exposure can induce measurable optical and surface changes in dental materials, although the magnitude of these effects depends on the material composition and treatment condition. In the individual specimens examined, acrylic resin showed wider EC distributions under several conditions, while the stainless-steel specimen showed comparatively narrower distributions in some treatment groups. Further studies using independently prepared replicate specimens are required to determine whether the observed patterns are reproducible and statistically representative of each dental material. For the Orahex-treated specimens, the observed correspondence between TD-OCT-derived extinction coefficients and NC-AFM roughness parameters suggests that changes in optical attenuation are associated with concurrent nanoscale surface modifications. Therefore, the extinction coefficient should be regarded as an integrated optical indicator of the biofilm–material interface rather than an exclusive measure of biofilm biomass or material degradation.

TD-OCT was demonstrated to be a sensitive, rapid, and non-destructive method for detecting subtle changes in dental materials, while NC-AFM provided detailed insights into nanoscale surface alterations. The integration of these techniques offers a comprehensive approach for assessing the long-term effects of oral care products on restorative materials and may contribute to the development of more durable and clinically reliable dental biomaterials. Future studies should include larger sample populations, longer exposure periods, and additional surface characterization techniques to further elucidate the mechanisms underlying material degradation and optical property changes.

## Figures and Tables

**Table 1:** Sample Size and Group Allocation of substrates used.

Group	Imaging Phase	Description	Sample Size	Contents
1	I only	Control set in BHI broth	3 *	Sterilized BHI Broth
		Inoculated samples in BHI broth	3 *	Inoculated BHI broth
		Sub-total	6	
2	II	Sonicated with Water	3 *	Water
	I	Inoculated samples in BHI broth	3 *	Inoculated BHI broth
		Sub-total	6	
3	II	Sonicated with Watsons	3 *	Watsons
	I	Inoculated samples in BHI broth	3 *	Inoculated BHI broth
		Sub-total	6	
4	II	Sonicated with Orahex	3 *	Orahex
		TOTAL	21	

* Comprising one acrylic resin, one composite resin, and one metal alloy specimen.

**Table 2:** Optical Thickness Derived from TD-OCT B-scan Images.

Treatment	Acrylic Resin (μm)	Composite Resin (μm)	Stainless Steel (μm)
Control	1.02 ± 0.04	0.94 ± 0.12	1.02 ± 0.03
P1 Water	1.05 ± 0.02	1.09 ± 0.15	0.94 ± 0.08
P1 Orahex	1.17 ± 0.04	1.13 ± 0.09	1.13 ± 0.11
P1 Watsons	1.09 ± 0.13	1.05 ± 0.21	1.24 ± 0.03
P2 Water	1.09 ± 0.21	1.17 ± 0.11	1.17 ± 0.07
P2 Orahex	1.17 ± 0.07	0.86 ± 0.06	1.13 ± 0.12
P2 Watsons	0.98 ± 0.12	1.02 ± 0.12	0.95 ± 0.02

## Data Availability

The raw data supporting the conclusions of this article will be made available by the authors on request.

## Supplementary Materials

The following supporting information can be downloaded at: https://www.mdpi.com/article/10.3390/dj14090563/s1, Supplementary File S1: Dental OCT.

## Abbreviations

The following abbreviations are used in this manuscript:

TD-OCT	Time Domain-Optical Coherence Tomography
NC-AFM	Non-Contact—Atomic Force Microscopy
EC	Extinction Coefficient
BHI	Brain Heart Infusion
CFU	Colony-Forming Unit
SEM	Scanning Electron Microscope

## References

[1] Baiju R.M., Peter E., Varghese N.O., Sivaram R. (2017). Oral Health and Quality of Life: Current concepts. J. Clin. Diagn. Res..

[2] Tadin A., Guberina R.P., Domazet J., Gavic L. (2022). Oral Hygiene Practices and Oral Health Knowledge among Students in Split, Croatia. Healthcare.

[3] Marsh P.D. (2010). Microbiology of dental plaque biofilms and their role in oral health and caries. Dent. Clin. N. Am..

[4] Mokeem L.S., Willis L.H., Windsor L.J., Cook N.B., Eckert G., Gregory R.L. (2021). Combined effects of soft drinks and nicotine on streptococcus mutans metabolic activity and biofilm formation. J. Oral. Sci..

[5] Koka K.M., Pillarisetti P., Yasangi M.K. (2021). Dental plaque biofilm: Development, pathogenicity and Analysis. Int. J. Sci. Healthc. Res..

[6] Jar A.A., Khormi A.A., Al-Khamiss N.A., Alnaim A.A., Alharbi Z.F., Holba H.A., Alshamrani A.S., Khormi A.A., Alharbi A.S., Almutairi A.M. (2023). The effect of dentures on oral health and the quality of life. Int. J. Community Med. Public Health.

[7] Neppelenbroek K.H. (2015). The importance of daily removal of the denture biofilm for oral and systemic diseases prevention. J. Appl. Oral. Sci. Rev. FOB.

[8] Bukhari M.A., Aldossari A.M., Alyami I.M., Al Shari A.H., Al Huwaidi A.A., Alzaid S.Y., Alqahtani A.M., Musayri M.I., AlShahrani Y.M. (2022). Pontic design and its effects on the health of the gingiva. Int. J. Community Med. Public Health.

[9] Zheng S., Bawazir M., Dhall A., Kim H.E., He L., Heo J., Hwang G. (2021). Implication of Surface Properties, Bacterial Motility, and Hydrodynamic Conditions on Bacterial Surface Sensing and Their Initial Adhesion. Front. Bioeng. Biotechnol..

[10] Yoda I., Koseki H., Tomita M., Takayuki S., Horiuchi H., Hideyuki S., Osaki M. (2014). Effect of surface roughness on biomaterials on *Staphylococcus* epidermidis adhesion. BMC Microbiol..

[11] Shah N., Logani A., Bansal N. (2014). Recent advances in imaging technologies in dentistry. World J. Radiol..

[12] Machoy M., Seeliger J., Szyszka-Sommerfeld L., Koprowski R., Gedrange T., Woźniak K. (2017). The Use of Optical Coherence Tomography in Dental Diagnostics: A State-of-the-Art Review. J. Healthc. Eng..

[13] Aumann S., Donner S., Fischer J., Müller F. (2019). Optical Coherence Tomography (OCT): Principle and Technical Realization.

[14] Palumbo G., Pratesi R., Sampson D., Hillman T.R., Häder D.-P., Jori G. (2004). Optical Coherence Tomography, Lasers and Current Optical Techniques in Biology.

[15] Vermeer K.A., Mo J., Weda J.J.A., Lemij H.G., De Boer J.F. (2013). Depth-resolved model-based reconstruction of attenuation coefficients in optical coherence tomography. Biomed. Opt. Express.

[16] Giessibl F. (2003). Advances in Atomic Force Microscopy. Rev. Mod. Phys..

[17] Tsenova-Ilieva I., Karova E. (2020). Application of atomic force microscopy in dental investigations. Int. J. Sci. Res..

[18] Aquino M., Mota C., Santos J., Nascimento P., Campello S., Gomes A. (2019). Optical coherence tomography as a tool to visualize biofilm formation over removable prosthesis. Proceedings of the SPIE 11078 Optical Coherence Imaging Techniques and Imaging in Scattering Media.

[19] Li H., Liu H., Zhang L., Hiewy A., Shen Y. (2023). Evaluation of extracellular polymeric substances matrix volume, surface roughness and bacterial adhesion property of oral biofilm. J. Dent. Sci..

[20] Cai J., Jung J., Dang M., Kim M., Yi H., Jeon J. (2016). Functional Relationship between Sucrose and a Cariogenic Biofilm Formation. PLoS ONE.

[21] Saravia M.E., Nelson-Filho P., Silva R.A.B., De Rossi A., Faria G., Silva L.a.B., Emilson C. (2013). Recovery of mutans streptococci on MSB, SB-20 and SB-20M agar media. Arch. Oral. Biol..

[22] Goto H., Cadondon J., Galvez M.C., Vallar E., Shiina T. (2025). OCT analysis of white clover leaves affected by regional ozone stress. Sci. Rep..

[23] Cadondon J., Vallar E., Galvez M.C., Shiina T. (2025). Microstructure, Extinction Coefficient, and Chlorophyll Content of Philippine Bamboo Leaves by a Portable TD-OCT Scanner. Proceedings of the 13th International Conference on Photonics, Optics and Laser Technology-PHOTOPTICS.

[24] Cadondon J., Goto H., Okaneya S., Shiina T. OCT Characterization and Spectral Chlorophyll Index Measurement of Plant Leaves During Senescence. Proceedings of the 14th International Conference on Photonics, Optics and Laser Technology.

[25] Cadondon J.G., Halapit H.A., Galvez M.C., Shiina T., Vallar E. (2026). Non-invasive assessment of bamboo leaf optical properties and microstructural changes under light stress using time-domain OCT. Sci. Rep..

[26] Baykara M., Schwarz U. (2016). Atomic Force Microscopy: Methods and Applications. Encycl. Spectrosc. Spectrom..

[27] Wang C., van der Mei H.C., Busscher H.J., Ren Y. (2020). *Streptococcus mutans* adhesion force sensing in multi-species oral biofilms. npj Biofilms Microbiomes.

[28] Damodaran N., Ramamurthy S., Velusamy S., Kanakaraj Manickam G. (2012). Speckle Noise Reduction in Ultrasound Biomedical B-Scan Images Using Discrete Topological Derivative. Ultrasound Med. Biol..

[29] Sukhadia A., Rohlfing D., Johnson M., Wilkes G. (2002). A comprehensive investigation of the origins of surface roughness and Haze in polyethylene blown films. J. Appl. Polym. Sci..

[30] Juhim F., Pien Chee F., Awang A., Salleh K.A.M., Ibrahim S., Hasim H., Rumaling M.I., Alrowaili Z., Al-Buriahi M. (2026). Optimization of rice husk fibre size fr improved density, transparency, and electron shielding efficiency in tellurite glass systems. J. Sci. Adv. Mater. Devices.

[31] Chiarelli A.M., Low K.A., Maclin E.L., Fletcher M.A., Kong T.S., Zimmerman B., Tan C.H., Sutton B.P., Fabiani M., Gratton G. (2019). The Optical Effective Attenuation Coefficient as an Informative Measure of Brain Health in Aging. Photonics.

[32] Moyal J., Dave P.H., Wu M., Karimpour S., Brar S.K., Zhong H., Kwong R.W.M. (2023). Impacts of Biofilm Formation on the Physicochemical Properties and Toxicity of Microplastics: A Concise Review. Rev. Environ. Contam. (Former. Residue Rev.).

[33] Fern C.-L., Liu W.-J., Chang Y.-H., Chiang C.-C., Chen Y.-T., Lu P.-X., Su X.-M., Lin S.-H., Lin K.-W. (2023). Surface Roughness-Induced Changes in Important Physical Features of CoFeSm Thin Films on Glass Substrates during Annealing. Materials.

[34] Valagiannopoulos C., Kovanis V. (2025). Watson transform in quantum scattering. Sci. Rep..

[35] Sauer K., Stoodley P., Goeres D.M., Hall-Stoodley L., Burmølle M., Stewart P.S., Bjarnsholt T. (2022). The biofilm life cycle: Expanding the conceptual model of biofilm formation. Nat. Rev. Microbiol..

[36] Zayed S.M., Aboulwafa M.M., Hashem A.M., Saleh S.E. (2021). Biofilm formation by *Streptococcus mutans* and its inhibition by green tea extracts. AMB. Expr..

[37] Valde D., Kumar T., Jujjavarapu S.E. (2026). Real-time sensing approaches and antibiofilm strategies for biofilm-associated infections: Advances and perspectives on medical devices and clinical contexts. Future J. Pharm. Sci..

[38] Kelly J.R. (2004). Dental ceramics: Current thinking and trends. Dent. Clin. N. Am..

[39] Ouajani K., Steck J.E., Olivares G. (2025). Leveraging Machine Learning for Porosity Prediction in AM Using FDM for Pretrained Models and Process Development. Materials.

[40] Xi C., Marks D., Schlachter S., Luo W., Boppart S.A. (2006). High-resolution three-dimensional imaging of biofilm development using optical coherence tomography. J. Biomed. Opt..

[41] Janjua O.S., Jeelani W., Khan M.I., Qureshi S.M., Shaikh M.S., Zafar M.S., Khurshid Z. (2023). Use of optical coherence tomography in dentistry. Int. J. Dent..

[42] Bhat S.V., Price J.D.W., Dahms T.E.S. (2021). AFM-based correlative microscopy illuminates human pathogens. Front. Cell. Infect. Microbiol..

[43] Rahnenführer J., De Bin R., Benner A., Ambrogi F., Lusa L., Boulesteix A.-L., Migliavacca E., Binder H., Michiels S., Sauerbrei W. (2023). Statistical analysis of high-dimensional biomedical data: A gentle introduction to analytical goals, common approaches and challenges. BMC Med..

[44] Welch K., Cai Y., Strømme M. (2012). A Method for Quantitative Determination of Biofilm Viability. J. Funct. Biomater..

