# Supplementary material for: Optical Attenuation Analysis of Biofilm Formation on Dental Prostheses Using Time-Domain Optical Coherence Tomography and Atomic Force Microscopy
Source: Dent J (Basel). 2026 Sep 3;14(9):563. doi: 10.3390/dj14090563 (PMC13605691; doi:10.3390/dj14090563)
Supplement: Supplementary file 1 [file dentistry-14-00563-s001.zip › dentistry-4406027-supplementary.pdf]

Optical Attenuation Analysis of Biofilm Formation on Dental Prostheses Using Time-Domain Optical Coherence Tomography and Atomic Force Microscopy

Maria Cecilia Galvez[1], Aaron Mharl Barlisan[1], Emmanuelle Hermosilla[1], Mark Nickole Tabafa[1], Ernest A. Macalalad[2], Jose Esmeria Jr.[3], Edgar Vallar[1], Seiroh Okaneya[4,6], Jumar Cadondon[1,4,5*], and Tatsuo Shiina[4]

[1]Environment And RemoTe sensing researcH (EARTH) laboratory, Department of Physics, College of Science, De La Salle University 0922, Manila, Philippines; maria.cecilia.galvez@dlsu.edu.ph (M.C.G.); aaron.brlsn@gmail.com (A.M.B.); msellehermosilla@gmail.com (E.H.); edgar.vallar@dlsu.edu.ph (E.V.)

[2]Interdisciplinary Space Missions Development Division, Space Science Missions Bureau, Philippine Space Agency, Metro Manila, Philippines; emacalalad@philsa.gov.ph (E.M.)

[3]Central Instrumentation Facility, Office of the Vice President for Research and Innovation, De La Salle University, Laguna Campus, Biñan City, Laguna, 4024, Philippines; jose.esmeria@dlsu.edu.ph (J.E.)

[4]Graduate School of Engineering, Chiba University, 1-33 Yayoicho, Chiba, Japan; shiina@faculty.chiba-u.jp (T.S.)

[5]Division of Physical Sciences and Mathematics, College of Arts and Sciences, University of the Philippines Visayas, Miagao, Iloilo 5023, Philippines

[6]Phoenix-Dent Co., Ltd., 2649-3, Kameicho, Sano-chi, Tochigi, 327-0024, Japan

*Corresponding author: jgcadondon@up.edu.ph

Supplementary File A.

1. **Control - Mean ECs**

AR	CR	SS
30.94148	28.81433	9.820369
24.93286	26.10897	29.70493
54.56802	27.76202	30.77378
14.34729	40.34546	45.20359
25.52952	34.45039	31.96835
23.46123	6.348642	43.16963
28.59128	34.34154	25.07478
20.62334	11.93914	44.16042
33.5876	39.32655	26.22275
35.11819	39.57184	31.90507
28.39208	8.853799	50.47697
20.36056	16.4617	
24.22582	4.790018	
27.68915	23.29291	
31.95826	21.77938	
27.39018	29.37252	
13.10199	35.51446	
18.63742	46.86555	
11.88686	31.98341	
22.99064		
20.99321		
15.06964		
29.19998		
19.98658		
17.63425		
22.89886		
40.77761		
43.14267		
16.54193		
15.43955		
45.96681		
37.18518		
38.97282		
26.73873		

2. Acrylic Resin - Mean ECs

PHASE 1				PHASE 2		
Water	Orahex	Watsons		Water	Orahex	Watsons
23.47122	43.4978	39.13409		95.07581	53.41865	33.62686
28.57538	88.51625	48.1218		13.11103	23.94936	43.89534
45.40977	41.6321	32.53177		56.32606	28.73318	43.24833
36.52183	38.46111	30.47259		38.75848	19.50558	37.29744
125.6084	32.83879	18.31938		33.6601	25.86041	23.64698
50.43414	20.56386	37.03528		49.94377	19.78857	26.11959
33.973	19.35336	33.54317		42.11427	46.78915	30.12737
34.82922	22.29677	43.59373		52.61051	32.436	30.64109
38.84812	25.7689	44.23205		42.94885	38.33277	38.77067
30.83701	31.2002	33.22018		40.13671	39.16918	38.139
57.95724	25.16866	22.20287		32.11238		31.42141
32.80523	32.85505	45.71753		32.50159		31.32786
31.4194	31.81476	37.60994		39.60008		46.08368
45.31197	43.67422	40.04999		33.99014		23.04937
36.52493	20.70451	29.86698		34.40349		32.28834
38.51669	32.51483	40.6001		23.01074		
35.26991	32.04078	29.89394		37.11702		
56.06725	22.87187	36.87499		34.0469		
38.07826	27.57959	41.99884		33.8832		
21.93366	30.187	40.5669		38.53389		
34.21939	36.89501	35.2371		33.91734		
33.67007	38.15908	24.2984		39.31493		
79.26355	66.83386	48.04749		41.20261		
24.99756	34.16387	34.49877		42.49562		
10.16858	28.58186	22.55264		37.44057		
42.4625	33.62307	26.53222				
37.43523	53.67195	34.11308				
42.64709	44.15928	30.00956				
39.19217	53.53282	35.78599				
47.63821	43.4978	49.93638				
	88.51625	31.65462				
	41.6321	57.73682				
	38.46111	34.96263				
	32.83879					

3. Composite Resin - Mean ECs

PHASE 1			PHASE 2		
Water	Orahex	Watsons	Water	Orahex	Watsons
28.11422	25.94657	31.15636	39.6658	16.59457	106.8882
9.794293	41.8721	35.22016	23.86515	12.52162	20.15423
13.77674	38.7351	34.08401	28.02267	41.15306	20.6687
21.66275	33.35366	19.94211	23.40707	21.72615	22.40894
31.53986	26.24139	55.54092	25.04986	38.21186	24.66979
36.39735	36.86508	50.37729	31.02664	43.89383	24.61895
33.30024	30.00894	33.85287	32.54056	37.03574	20.10057
37.87924	40.15967	37.13252	35.58141	28.12075	19.06153
24.14545	26.34027	30.89113		41.2592	17.71773
33.16589	28.64905	44.49297		13.28093	15.38993
26.39965	40.71956	33.28166			
25.80449	31.31502	39.3601			
	38.85556	41.24743			
	30.23268	42.12687			
	41.93637	40.99026			
	144.5637	53.78435			
	30.52112				

4. Stainless Steel - Mean ECs

PHASE 1			PHASE 2		
Water	Orahex	Watsons	Water	Orahex	Watsons
18.3316	36.27142	37.22252	42.05578	39.32453	46.84
33.08648	30.75293	31.79717	34.05953	48.1808	45.68576
15.87965	35.85723	38.12435	41.23091	37.98307	48.39225
26.77812	36.70874	40.20977	32.11427	34.589	87.83641
37.32972	23.83481	19.20151	30.50801	25.69532	33.38127
21.10454	20.41861	37.31671	36.85665	30.79556	42.27504
29.03222	19.94976	35.81722		38.60065	48.52052
25.82355	16.64547	33.46822		54.62397	
31.63243	23.93575	37.56785		40.56556	
27.76946	27.9859	40.31674		36.1505	
9.433919	43.22617			64.81999	
30.53345					
47.19496					

Supplementary File B. Data and Program

https://drive.google.com/drive/folders/1oCL800pM25Nqcx3YfleIOlF08hB7DxIM?usp=drive_link
